# Response to low dose growth hormone treatment in infants and toddlers with Prader-Willi Syndrome

**DOI:** 10.1186/1687-9856-2015-S1-O31

**Published:** 2015-04-28

**Authors:** Elly Scheermeyer, Mark Harris, Ian Hughes, Patricia A Crock, Geoffrey Ambler, Charles F Verge, Phil Bergman, George Werther, Maria E Craig, Catherine S Choong, Peter SW Davies

**Affiliations:** 1 Faculty of Medicine and Biomedical Sciences – School of Medicine, QCMRI, The University of Queensland, Brisbane, QLD, Australia; 2 Lady Cilento Children's Hospital, Brisbane, QLD, Australia; 3 MRI-UQ, The University of Queensland, Brisbane, QLD, Australia; 4 John Hunter Children’s Hospital, Newcastle, NSW, Australia; 5 School of Medicine and Public Health, University of Newcastle, Newcastle, NSW, Australia; 6 The Children’s Hospital at Westmead, Sydney, NSW, Australia; 7 Discipline of Paediatrics and Child Health, The University of Sydney, Sydney, NSW, Australia; 8 Sydney Children’s Hospital, Randwick, NSW, Australia; 9 School of Women’s and Children’s Health, University of New South Wales, Sydney, NSW, Australia; 10 Monash Medical Centre, Melbourne, VIC, Australia; 11 The Royal Children’s Hospital and Murdoch Children’s Research Institute, Melbourne, VIC, Australia; 12 Princess Margaret Hospital Perth, Perth, WA, Australia; 13 School of Pediatrics and Child Health, University of Western Australia, Perth, WA, Australia

## Aim

We aimed to assess the benefits and safety of low dose growth hormone treatment (GHT, 4.5 mg/m^2^/week) in young children with genetically confirmed Prader-Willi Syndrome (PWS).

## Methods

Data of 20 infants (2-12 months) and 24 toddlers (13-24 months) were collected from the PWS-OZGROW database. The two groups were evaluated for standard deviation scores (SDS) of height (length under age 2 years), weight and BMI using the World Health Organization standards (SDS_WHO_) and PWS specific BMI (SDS_PWS_), bone age (BA), insulin-like growth factor-1 (IGF-1) levels, hypotonia, developmental delay, spinal curvature, sleep studies and adverse events over 2 years of GHT.

## Results

At commencement of GHT infants had a reduced BMI SDS_WHO_ (P=0.003), while toddlers had a reduced height SDS_WHO_ (P=0.014). The height/length SDS_WHO_ of infants increased from -1.09±1.15 at baseline to -0.26±0.89 after one year and -0.02±0.80 after two years GHT (GLM repeated measures; P <0.0001,) and in toddlers increased from -2.11±1.45 to -1.11±1.11 and -0.87±0.94 (P <0.0001). BMI SDS_WHO_ increased in both groups (data not shown), while BMI SDS_PWS_ decreased (P<0.0001, age groups P>0.05) from 0.40±0.84 to -0.07±0.67 at Year 1 and -0.31±0.95 at Year 2 (both age groups combined). Preterm and full term children did not differ significantly in response to GHT, nor did children with deletion (14) and uniparental disomy (16). All children had low to very low serum IGF-1 at baseline which increased to within the normal reference range for the majority of children (61%) with the remainder modestly increased during the first 2 years of treatment. An improvement in tone, spinal curvature and developmental delay was noted in those who were more severely affected at baseline. Two children developed scoliosis. Three children ceased GHT temporarily to adjust positive airway pressure settings or for tonsillectomy following onset or worsening of obstructive and/or central sleep apnoea. Bone age was not advanced and no other serious adverse events were reported during the two year GHT.

## Conclusion

Treating young children (<2 yrs) with PWS with 4.5 mg/m^2^/week of GH normalises height and achieves IGF-1 levels in the normal range in the majority of patients. The risk of respiratory adverse events can be minimised by regular monitoring. The dose was sufficient to keep height and most IGF-1 values in the normal range and PWS specific BMI SDS in a negative range and may lower potential risks of long-term treatment of very young children with PWS.

On behalf of the PWS and OZGROW collaboration

